# Role of Lung Function Monitoring by the Forced Oscillation Technique for Tailoring Ventilation and Weaning in Neonatal ECMO: New Insights From a Case Report

**DOI:** 10.3389/fped.2018.00332

**Published:** 2018-11-01

**Authors:** Genny Raffaeli, Chiara Veneroni, Stefano Ghirardello, Anna Lavizzari, Sofia Passera, Fabio Mosca, Giacomo Cavallaro, Raffaele L. Dellacà

**Affiliations:** ^1^NICU, Fondazione IRCCS Ca' Granda Ospedale Maggiore Policlinico, Università degli Studi di Milano, Milan, Italy; ^2^Dipartimento di Elettronica, Informazione e Bioingegneria, Politecnico di Milano, Milan, Italy

**Keywords:** extracorporeal life support, mechanical ventilation, forced oscillation technique, lung mechanics, newborns, respiratory insufficiency

## Abstract

Respiratory management during extracorporeal membrane oxygenation (ECMO) is complex. Assessment of lung mechanics might support a patient-tailored ventilatory strategy. We report, for the first time, the use of the forced oscillation technique (FOT) to evaluate lung function during neonatal ECMO to improve the individualization of respiratory support. The patient was a formerly preterm infant at a corrected age of 40 weeks (gestational age 32 weeks) undergoing veno-arterial ECMO for refractory respiratory failure secondary to influenza A (H1N1) pneumonia. We used the FOT as a bedside non-invasive tool for daily monitoring of lung mechanics, from ECMO day 6 (E6) until decannulation. A small-amplitude, 5-Hz oscillatory pressure was overimposed on the ventilation waveform at the airway opening during positive end-expiratory pressure (PEEP) trials. From E6 to E9, lung mechanics changes with PEEP indicated a largely de-recruited and easily over-distendable lung that was not recruitable by applying lung-protective PEEP values. After surfactant and steroid administration, oscillatory reactance (Xrs) values began improving, suggesting a more recruited and pressure-recruitable lung. On E11, despite the lack of improvement in the radiographic appearance of the thorax, the FOT measurements showed a more recruited lung. Weaning from ECMO was started, and the patient was extubated within 48 h. The decannulation was successful, and the patient was extubated within 48 h after ECMO weaning. First-year respiratory and neurodevelopmental follow-up evaluation was unremarkable. This report suggests the potential usefulness of the FOT for monitoring the lung mechanics of ventilated newborns during ECMO to achieve individualized respiratory management. Such tailoring might improve neonatal outcomes and support clinicians with the establishment of a timely and safer weaning approach. These findings need to be verified on a larger population.

## Introduction

Extracorporeal membrane oxygenation (ECMO) is a life-saving procedure for refractory cardiorespiratory failure (1). Despite advances in neonatal care, ~600 newborns still require ECMO each year, worldwide (2). The primary aim is to provide native lung “rest,” thereby allowing time for healing (1). However, both the nature of this “rest” and optimal respiratory management during ECMO remain unclear (3).

Although higher positive end-expiratory pressures (PEEPs, 12–14 cmH_2_O) have been suggested to guarantee alveolar recruitment (4), lower PEEPs have also been adopted, thus contributing to high heterogeneity and lack of consensus in ventilator management (5). Moreover, the variability of lung conditions requires different responses to ventilation, necessitating a patient-tailored approach to avoid ventilator-induced lung injury (3). Another complex issue of ECMO is the timing of weaning: premature weaning may affect the overall patient outcome, whereas later decannulation may increase the risk of complications.

In current clinical practice, the native lung status of ventilated patients is investigated through continuous oxygen saturation monitoring, serial blood gas analysis, and thoracic radiography. However, these methods are only partially suitable during ECMO, because gas exchange is mainly determined through extracorporeal support, and thoracic radiography provides a static picture of the native lung. Alternatively, the measurement of lung mechanical properties offers the advantage of providing physicians with an informative background for setting the ventilator and monitoring the disease progress. Estimation of dynamic compliance has been proposed in the literature, but it requires a deeply sedated/paralyzed patient or the use of an esophageal balloon, making this approach clinically unsuitable in patients receiving ECMO. Moreover, data analysis is not straightforward, and the results are affected by intra-tidal recruitment and are dependent on breathing frequency (6).

The forced oscillation technique (FOT) is a non-invasive technique for measuring respiratory mechanics based on the application of a high-frequency and small amplitude oscillatory pressure overimposed on the ventilation waveform. From the relationship between the forcing pressure and the resulting oscillatory flow, the mechanical properties of the respiratory system can be estimated regardless of the presence of spontaneous breathing activities. The FOT has been validated for the assessment of lung volume recruitment and the identification of the optimal mechanical open-lung PEEP (7–10), and has been applied in animal models of prematurity (11) and mechanically ventilated preterm infants (12–14).

Here, we report the application of the FOT to individualize ventilatory management in challenging neonatal respiratory ECMO.

## Case report

This study was approved by the local ethics committee (Milan Area 2, Italy). Parents gave written informed consent for the publication of this case report in accordance with the Declaration of Helsinki.

A previous report describing this case was focused on the infectious disease perspective (15). Here we report on the use of the FOT for tailoring and monitoring the efficacy of the ventilation strategy. A female preterm infant at 40 weeks' postmenstrual age (gestational age 32^+3^ weeks, day of life 51) required veno-arterial ECMO for refractory hypoxemic respiratory failure, secondary to influenza A (H1N1) viral pneumonia. In combination with a 120-mL/kg/min venoarterial ECMO flow (Quadrox iD®, Maquet Getinge, Rastatt, Germany), synchronized intermitted positive pressure ventilation (Babylog® VN500, Drager, Lubeck, Germany) was initially adapted to “lung rest.” Peak inspiratory pressure was set at 20 cmH_2_O (from 35 to 38 cmH_2_O pre-ECMO); PEEP at 10 cmH_2_O (from 6 cmH_2_O); respiratory rate at 15/min (from 35/min); fraction of inspired oxygen at 0.21 (FiO_2_, from 1). Based on these parameters, effective tidal volumes were measured at ~1.5–2 mL (not weight-normalized). From ECMO day 1 (E1) to E5, the ventilatory setting was mainly tuned according to the daily thorax radiographic appearance, because both oxygenation and acid-base balance were maintained through extracorporeal support. However, because of the absence of clinical improvement, from E6, lung mechanics were characterized by repeated, bed-side FOT measurements performed by connecting the patient to a Fabian HFO ventilator (Acutronic CH, Bubikon, Switzerland) with software capable of performing FOT measurements and exporting pressure and flow data. From these data, we computed the total respiratory system reactance (Xrs) at 5 Hz, accounting for the elastic and inertial properties of the respiratory system (16). Xrs is related to lung compliance and accounts for both lung volume de-recruitment and lung (over)distention. Unfortunately, it is impossible to determine from a single Xrs measurement whether low Xrs values are due to de-recruitment or (over)distention and, because of the lack of proper reference measurements, it is difficult to assess whether an Xrs value is adequate for a specific infant.

Therefore, we evaluated Xrs during incremental/decremental PEEP trials because, by observing Xrs changes during this procedure, it is possible to obtain information about lung volume recruitability and distensibility with PEEP (12, 14) and to identify the PEEP providing the optimal compromise between alveolar recruitment and lung tissue stress (8–10).

These PEEP trials were designed for (1) probing the lung mechanical conditions by providing information regarding Xrs dependence on PEEP and (2) performing recruitment procedures (as a result of the relatively high PEEPs applied) and simultaneously evaluating their effectiveness.

Briefly, changes in Xrs with PEEP are mainly and jointly determined by lung recruitment/de-recruitment and increases/decreases in the degree of lung tissue stress. By comparing Xrs values at the same PEEP (i.e., the same distending pressure) during the increasing and the decreasing limbs of the PEEP trial, it is possible to determine whether the high PEEP values applied during the trial resulted in lung volume recruitment. The PEEP value corresponding to the maximal Xrs is the one providing the optimal mechanical balance maximizing lung volume recruitment with minimal tissue overdistention. A decrease in Xrs with increasing PEEP is suggestive of lung tissue distention prevailing over lung volume recruitment. The steeper the Xrs decrease with increasing PEEP, the higher the stress applied to lung tissues. A decrease in Xrs with decreasing PEEP is consistent with lung volume de-recruitment prevailing over the reduction in tissue distention. The steeper the Xrs decrease with reducing PEEP, the greater the instability of the lung periphery (i.e., the tendency to de-recruit). A graphical explanation of these concepts is provided in the Supplementary Material.

**Figure 1:**
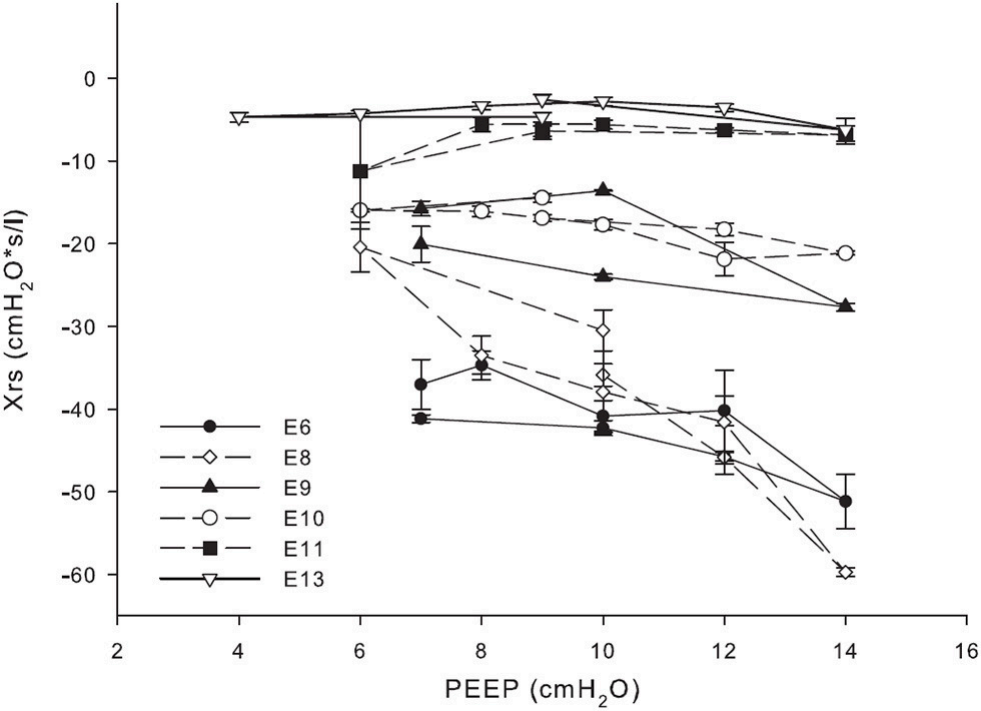
Relationship between reactance (Xrs) at different positive end-expiratory pressure (PEEP).

Table 1 shows the main respiratory setting and the diagnostic and therapeutical interventions, and Figure 1 shows the relationship between Xrs and PEEP values for the most representative trials. On E6, the thoracic radiographic examination revealed a “white lung.” The effective tidal volumes were suboptimal, with a pre-FOT trial PEEP set to 10 cmH_2_O (Table 1). The large negative values and the negative slope of the Xrs vs. PEEP graph were indicative of a massively de-recruited lung. The absence of hysteresis between the increasing and decreasing limbs of the trial suggested that even a PEEP of 14 cmH_2_O did not lead to significant alveolar recruitment (8–10).

**Table 1:** Main respiratory setting, diagnostic, and therapeutical interventions relevant to the ventilation monitoring and management of the patient.

**ECMO day**	**E6**	**E9**	**E11**	**E13**
Ventilation mode pre-FOT	AC/no VTG	AC/no VTG	AC/no VTG	AC+VTG
PIP (cmH_2_O)	25	25	25	26
PEEP pre-FOT (cmH_2_O)	10	7	8	9
PEEP post-FOT (cmH_2_O)	7	8	9	9
MAP (cmH_2_O)	13	11	18	14
RR set (bpm)	16	15	25	38
IT (sec.)	0,6	0,6	0,6	0,45
FiO2	0,4	0,60	0,40	0,40
Vt effective (mL)	1,6 - 4,8	5,30	8,70	15,00
SpO2 pre-FOT (mean)	94-95	96%	95-96%	95%
pH/pCO2/BE before	7,32/58/+2	7,38/69,5/+6,4	7,34/49,5/+1.1	7,46/31/−0,8
			
Blood flow/Sweep gas (ml/kg/min)	150/150	150/200	60/100	NA
FiO_2_ sweep gas (mean)	0.5	0.5	0.5	NA
**Technical issues**		**Weaning**
Chest X-ray	Bilateral patchy opacifications bilaterally severely hypodiaphan bilaterally hypodistended	Bilateral patchy opacifications + small right pleural effusion Bilaterally severely hypodiaphan Bilaterally hypodistended	Worsening patchy opacification in the right lung, stable pleural effusion Bilaterally normally distended	Same as E11
Forced Oscillation Technique	Rigid and massively derecruited lung easily distended and not recruitable within the tested pressure range	Rigid lung recruitable within the tested pressure range	More compliant lung, derecruitment at the lowest peep tested	More compliant lung at all the explored PEEP
Relevant therapeutical interventions	SURF 200 mg/kg	E8: SURF (200 mg/kg) E9: BAL with SURF (73 mg/kg) E9: Start DEX 0.25mg/kg twice/d chest physiotherapy SLI, PEEP trial	E10: DEX 0.25mg/kg once/d E11:SURF 200mg/kg + DEX 0.125mg/kg twice/d chest physiotherapy SLI, PEEP trial	E12: DEX 0.125 mg/kg twice/d chest physiotherapy SLI, PEEP trial
Sedoanalgesia μg/kg/h	MOR 100 + MID 180	MOR 100 + MID 180	MOR 100 + MID 180	MOR 100 + MID 180
Extra bolus MOR/MDZ during FOT	2/2	1/1	3/1	0/2
Rocuronium mg/kg/die	2,4	2,4	2,4	0

*BAL, bronchoalveolar lavage; bpm, breath per minute; FiO_2_, fraction of inspired oxygen; IT, inspiratory time; MAP, mean airway pressure; MOR, morphine; MID, midazolam; PIP, peak inspiratory pressure; PEEP, positive end-expiratory pressure; SLI, sustained lung inflation; SURF, surfactant; DEX, dexamethasone; RR, respiratory rate; Vt, tidal volume*.

Therefore, we reduced the PEEP to a minimum value of 7 cmH_2_O because greater pressures were not providing any benefits in terms of lung volume recruitment and were unnecessarily exposing lung tissue to mechanical stress, as suggested by the marked Xrs decrease with PEEP. On E8, Xrs continued to indicate the absence of lung volume recruitment, despite a very limited improvement (i.e., increase) in Xrs value at the lowest PEEP. Therefore, we administered surfactant (200 mg/kg endotracheally) and started chest physiotherapy through thoracic percussions, vibrations, and gentle bagging. PEEP trials and sustained lung inflations were performed, as clinically relevant. The following day (i.e., E9) Xrs improved, showing less-negative values and a less-pronounced negative slope with increasing PEEP, suggesting a more recruited lung. Furthermore, increased hysteresis between the increasing and decreasing PEEP limbs of the trial suggested that the lung was now recruitable, with an optimal PEEP of ~10 cmH_2_O because (1) at this PEEP, both Xrs in the decreasing limb and its difference from the corresponding Xrs in the increasing limb were maximal, and (2) further reducing PEEP below this pressure leads to alveolar de-recruitment, as evidenced by the reduction in Xrs.

At E10, despite no evidence of radiological improvement, Xrs demonstrated further slight improvement, showing a reduced negative slope with increasing PEEP. In addition, the reduced dependency of Xrs on PEEP suggested a more stable lung. Because of the appearance of clots in the venous side of the oxygenator, combined with the worsening of hemostatic test results, which were suggestive of an early stage of disseminated intravascular coagulation, a fast and successful decannulation was required. Based on the substantial functional improvement after the surfactant was administered and the clinical need to anticipate the weaning process, we started dexamethasone intravenously (0.25 mg/kg 2 times/day) and we performed broncho-alveolar lavage with diluted surfactant (1:2 dilution; 75 mL/kg porcine surfactant Curosurf ®, Chiesi Farmaceutici SpA, Parma, Italy) followed by a lung volume recruitment procedure. Based on the “open lung” concept, our aim was to set the operating lung volumes at the lowest values preventing lung volume de-recruitment in the deflation limb of the pressure/volume curve. We also performed incremental/decremental PEEP trials by monitoring the oxygenation status (17). On E11, the FOT results showed an improved lung recruitment overall, with some residual instabilities (e.g., lower Xrs values with higher std) at lower PEEPs (< 8 cmH_2_O). On the same day, because of the enhanced clinical conditions and overall encouraging FOT results, weaning was started using stepwise reduction of blood flow over a 24-h period. PEEP levels were maintained at 9–10 cmH_2_O throughout the weaning process, whereas the respiratory rate was gradually increased. The last surfactant dose (200 mg/kg) was administered to further optimize lung mechanics, whereas the steroid therapy was progressively stepped down. Once the blood flow was < 30 mL/kg (0.1 Lpm), after a short period of observation, the circuit was clamped and the cannulae were surgically removed on E13. On the same day, Xrs indicated a stable and recruited lung at all investigated PEEPs. The patient was successfully extubated 48 h after ECMO decannulation. She required another 20 days of non-invasive ventilation before being discharged with spontaneous breathing and no need for supplemental oxygen. Her first year respiratory and neurodevelopmental follow-up evaluation was unremarkable.

## Discussion

This report focused on the monitoring of lung mechanics during incremental/decremental PEEP trials in a ventilated formerly preterm infant receiving ECMO for severe respiratory failure. As is commonly acknowledged, ECMO may be temporarily established for two main reasons: first, for rescue oxygenation; second, to reduce—ventilator-induced harm. Ventilation parameters are reduced soon after extracorporeal bypass starts, to buy time for the lung to heal (1, 3, 5). However, this approach is widely variable and poorly supported by respiratory function monitoring in clinical practice (5). During ECMO, lung compliance may vary rapidly, and the uncontrolled up- or down-tuning of ventilation settings may result in lung volume de-recruitment or overdistention (3).

In our case, the FOT assessment allowed PEEP to be optimized and the effectiveness of “open lung” procedures (18) to be monitored according to the underlying pathophysiological features of the patient, which varied widely in the different phases of the infection. Both pneumonia itself and the ECMO-related systemic inflammatory response syndrome may have contributed to lung inflammation, thus impairing native lung compliance. In this context, the FOT allowed the effects of the anti-inflammatory interventions and concurrent spontaneous healing to be monitored with timely identification of improvements to the lung mechanics. In this respect, thoracic radiography proved to be less informative because radiological appearance did not improve as fast or as much as the lung mechanics did.

Another critical phase of ECMO is the weaning process, which depends on the adequate recovery of cardiopulmonary function. Although the decision to disconnect a patient from the extracorporeal circuit is traditionally supported by adequate radiographic recruitment, gas exchange, ventilator settings, and echocardiographic findings (1), the monitoring of lung mechanics may provide a team with further information that improves the timeliness and confidence of weaning decision-making. Moreover, a previous study reported that measuring pulmonary mechanics helps in predicting successful weaning from ECMO (19). In conclusion, FOT allows bedside monitoring of lung mechanics in clinical practice and, when combined with common evaluations such as blood gas analysis and thoracic radiography, it may (1) provide useful information for a patient-tailored respiratory support strategy for ECMO patients and (2) support clinicians in evaluating disease progression and identifying the optimal timing for ECMO weaning, thereby reducing the risk of premature or late decannulation, which can both lead to adverse outcomes. Future studies should validate the use of the FOT for the clinical management of these patients.

## Author contributions

GC, GR, RD, CV, and FM contributed conception and design of the study. GR, GC, CV, and RD wrote the first draft of the manuscript. SG, AL, and FM provided extensive critical revision. All authors contributed to manuscript critical revision, read, and approved the submitted version.

### Conflict of interest statement

Politecnico di Milano University, Institution of RD and CV, owns a patent on the use of FOT for optimizing PEEP. Politecnico di Milano received a research grant from Chiesi Farmaceutici, Philips Respironics, and Acutronic. The remaining authors declare that the research was conducted in the absence of any commercial or financial relationships that could be construed as a potential conflict of interest.

## Abbreviations

E (day): ECMO (day)
ECMO: extracorporeal membrane oxygenation
FOT: forced oscillation technique
PEEP: positive end-expiratory pressure
Xrs: reactance.
