# Supplementary material for: Role of Lung Function Monitoring by the Forced Oscillation Technique for Tailoring Ventilation and Weaning in Neonatal ECMO: New Insights From a Case Report
Source: Front Pediatr. 2018 Nov 1;6:332. doi: 10.3389/fped.2018.00332 (PMC6221953; doi:10.3389/fped.2018.00332)
Supplement: Supplementary file 1 [file Presentation_1.PPTX]

## Slide 1
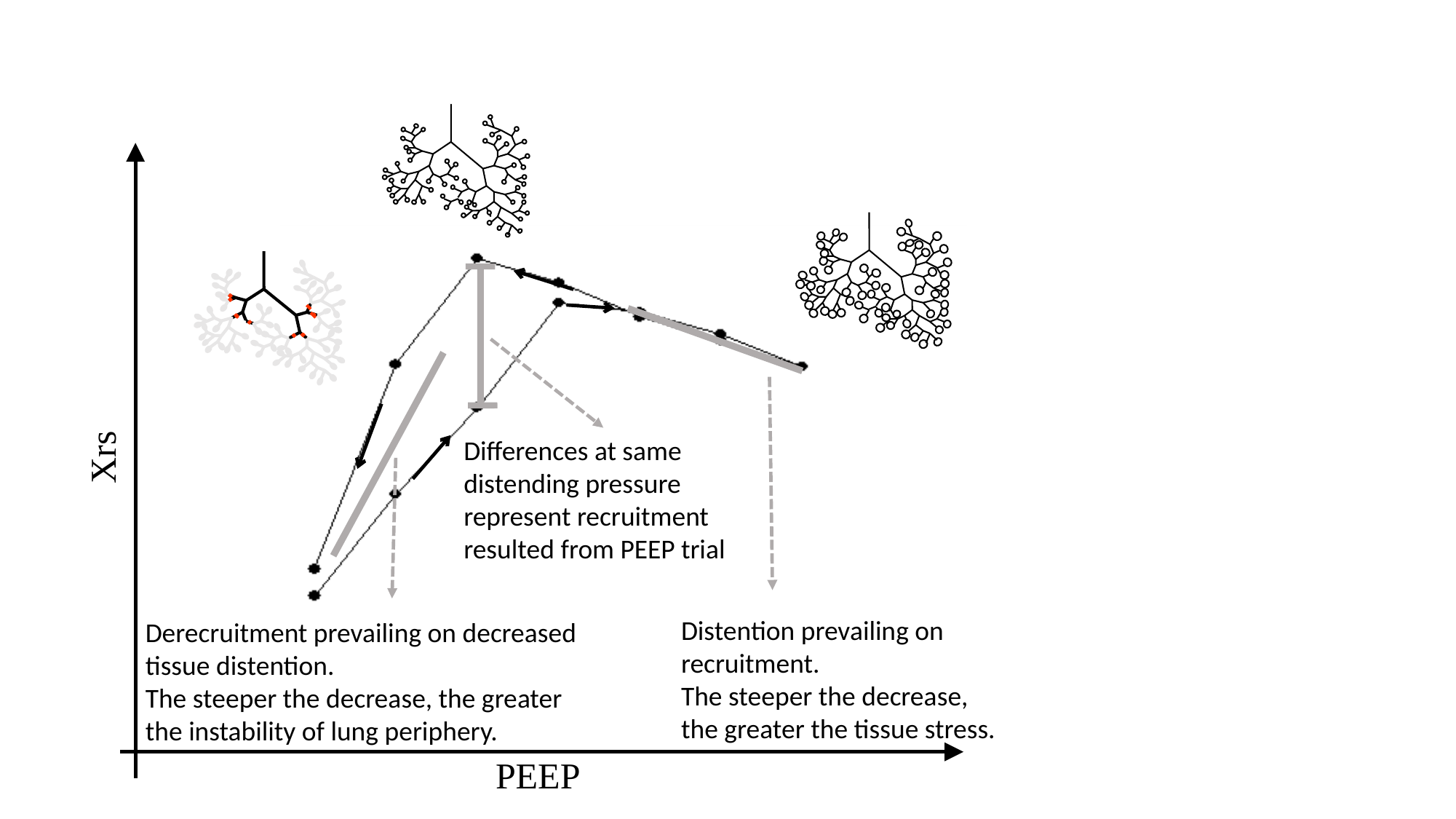

Differences at same
distending pressure represent recruitment resulted from PEEP trial
Xrs
Distention prevailing on recruitment.
The steeper the decrease, the greater the tissue stress.
Derecruitment prevailing on decreased tissue distention.
The steeper the decrease, the greater the instability of lung periphery.
PEEP
